# Difference in force direction during propulsion between subjects with different push-off locations

**DOI:** 10.1186/1757-1146-3-S1-P8

**Published:** 2010-12-20

**Authors:** Björn Englund, Toni Arndt

**Affiliations:** 1 Karolinska Institute, Stockholm, Sweden

## 

**Figure 1:**
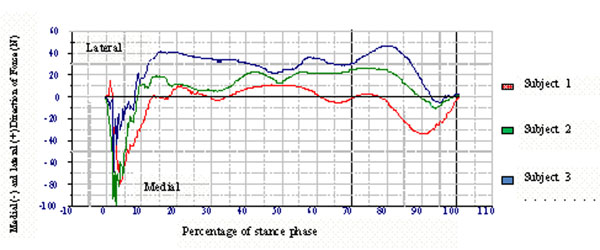


It could be hypothesized that when walking in a linear direction, the most efficient strategy would be to focus the force created by the push-off in the absolute opposite direction to the direction of propulsion. Forces in a medial or lateral direction could then be considered as loss of efficiency since these forces do not create movement in the intended direction. The aim of this study was to investigate if individual differences in push-off location of the forefoot resulted in difference in force direction. Three subjects with different push-off locations were identified through video gait analysis. The subjects were categorized as having either a lateral (subject 1), central (subject 2) or medial (subject 3) push of location. The subjects walked over a force plate (Kistler) where medial and lateral forces were registered. The results showed distinct differences in force direction between the subjects during the propulsive period of gait (70%-100%) (see figure 1).

Difference in force direction may lead to differences in propulsive efficiency. It could also be hypothesized that proximal segments need to compensate in order to keep a subject with medial or lateral force deviations walking in a linear direction of propulsion.

